# Aspects of the ecology of the earthworm *Eisenia lucens* (Waga 1857) studied in the field and in laboratory culture

**DOI:** 10.1007/s11356-019-06187-7

**Published:** 2019-08-27

**Authors:** Joanna Kostecka, Kevin R. Butt, Anna Mazur-Pączka, Grzegorz Pączka, Mariola Garczyńska, Agnieszka Podolak

**Affiliations:** 1 Faculty of Biology and Agriculture, Department of Natural Theories of Agriculture and Environmental Education, University of Rzeszów, 1a Cwiklinskiej St., 35-959 Rzeszow, Poland; 2 University of Central Lancashire, Forensic and Applied Sciences, Preston, PR1 2HE UK

**Keywords:** Cocoon, Cultivation, Dead wood, Dentario glandulosae-Fagetum, Growth, Hatchling, Reproduction, Seasonal dynamics

## Abstract

This work relates data from field sampling of *Eisenia lucens* and from laboratory-based culture. Field sampling used soil sorting and vermifuge extraction and took place in beech-dominated forests of southwest Poland. Initial work derived population estimates from four sub-communities of the forest looking for seasonal dynamics and later work employed targeted sampling in summer within rotting wood to obtain live specimens for laboratory culture. A preliminary examination within and below rotten wood during winter was also undertaken. In the laboratory, clitellate earthworms were kept at 20 °C, the substrate changed every 6 months, and the population examined. Cocoons were incubated individually at 15 °C, with number of hatchlings per cocoon and the mass of each determined. Hatchlings were grown at 15 °C in field-collected wood and compared with growth in a 1:1 volume ratio of wood and horse manure. Further hatchlings were fed with horse manure only (at 10 °C) and after 19 weeks, half were transferred to 15 °C. In the field, mature individuals varied significantly (*p* < 0.01) in biomass between 2 sampling sites where found, with an overall mean density across sites of 4.14 ± 3.53 m^−2^ with a mean biomass of 2.21 ± 1.93 g m^−2^. Numbers in soil varied over the sampling period, with a suggestion that this species moves from mineral soil to organic-rich dead wood as conditions permit. In summer, all life stages were recovered from rotting wood above the mineral soil. Sampling in winter found cocoons in rotting wood below snow. These hatched rapidly (within 2 weeks) when taken to the laboratory. Laboratory culture allowed maintenance of a population for 2 years. Mean cocoon mass was 50.6 mg with a mean of 2.9 hatchlings per cocoon and hatchling mass was inversely proportional to number per cocoon. Growth with 50% horse manure was significantly greater (*p* < 0.001) than with wood. Increased temperature from 10 to 15 °C brought more significantly (*p* < 0.05) rapid growth. To culture this species through its life cycle, a natural substrate is needed, but then it is necessary to acclimate the animals to something more easily obtainable. More work is needed from field sampling to fully understand the seasonal dynamics of this species, which utilises different parts of the soil profile throughout the year.

## Introduction

*Eisenia lucens* (Waga 1857) is a Lumbricidae earthworm, having a specific body colour characterized by alternating dark red-brown and lighter off-yellow bands along the full length of its body. This species has a relatively narrow geographical range in Central and Eastern Europe, typically inhabiting montane forest habitats of the Carpathian Mountains (Szederjesi et al. 2018). *E. lucens* can commonly be located above the soil surface, under bark and in damp decomposing wood of fallen trees and stumps (Pes et al. 2016). Due to a large body size compared with other species of earthworms living in litter and rotting tree trunks in mountain forests (Plisko 1973; Kasprzak 1986), it plays a major role in the re-distribution of woody resources in these ecosystems.

**Fig. 1:**
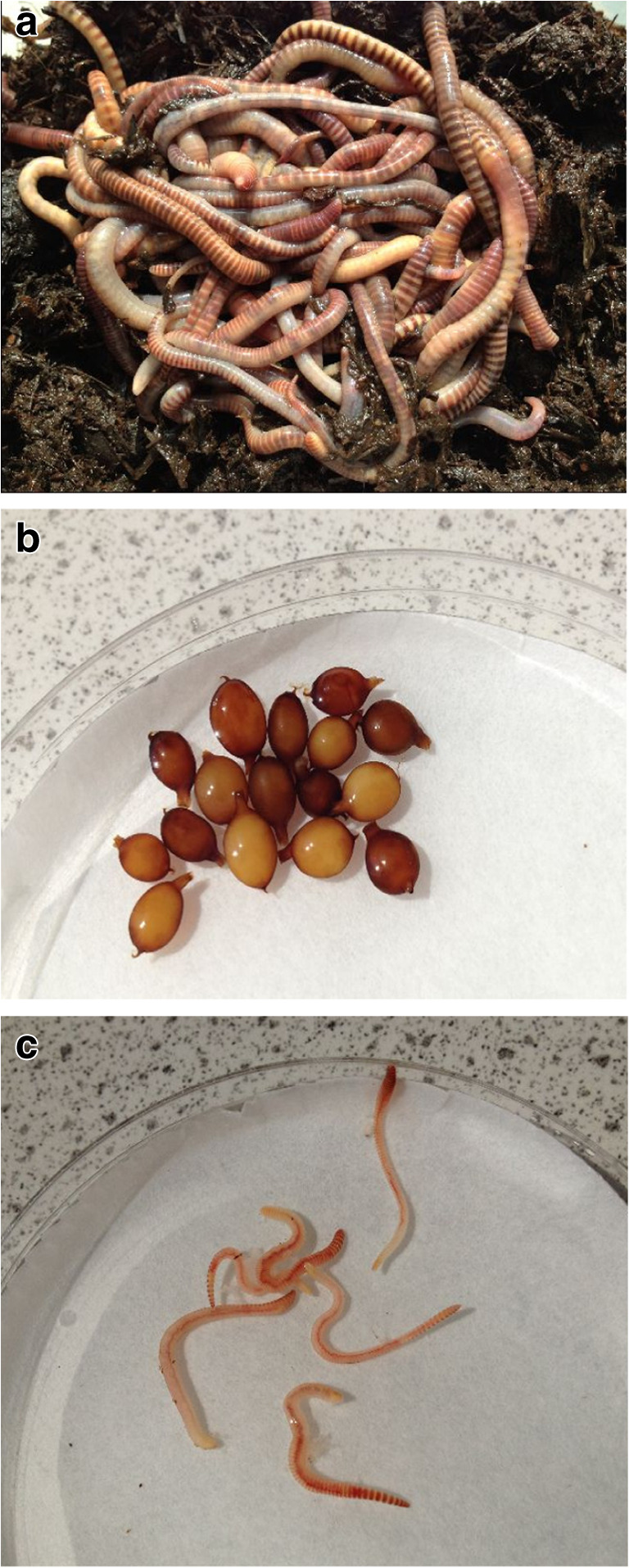
Life stages of *E. lucens* **a** adults (0.9–2.2 g); **b** cocoons (approx. 50 mg); **c** hatchlings (5–35 mg). (Cocoons and hatchlings are shown within 9 cm dia. Petri dishes)

Unpublished data (Kostecka 1988), from beech-dominated sites at 700–950 m elevation in the Bieszczady Mountains (Poland), showed that this species also inhabits the mineral soil at specific times of the year. This field data is now presented along with a preliminary investigation undertaken when snow was lying in the mountain forests (January 2019). In addition, to gather basic information on all life stages (adults, hatchlings and cocoons; Fig. 1) earthworms were kept under laboratory conditions. Similar work has been undertaken with *Allolobophora carpathica* and with *Dendrobaena alpina* (Kostecka and Butt 2001, 2015) obtained from the same geographical location.

One aim of this research was therefore to bring together field data from earlier (1980s) investigations, from summer collections and from a more recent exploratory visit in winter to consider the seasonal dynamics of this species. It is suggested that vertical movements from rotting wood above the soil surface, to depths in the mineral soil may be occurring in winter. A further aim was to collect specimens of *E. lucens* to gather selected fundamental biological information relating to, e.g. effects of temperature and feed stock on life stages and determine if this species could be cultured under laboratory conditions.

## Material and methods

### Fieldwork

Field sampling in 1986 (July to November) and 1987 (May to November) was undertaken at sites within the Bieszczady National Park (BNP) in beech forest (Dentario glandulosae-Fagetum). Sampling outside of these months was prevented by seasonal heavy snowfall at altitudes above 700 m. Four phytosociological groups (sub-assemblies) of the woodland (see Table 1 for details) were sampled using the method of Zajonc (1970) with a combination of hand-sorting of soil followed by formalin (0.4%) extraction in the hole created. Soil samples were 0.25 m × 0.25 m (*n* = 10 per site), dug to 0.25 m. Soil environmental data, such as temperatures, were also collected. All animals collected in the field were preserved in 4% formalin for later identification (Plisko 1973; Kasprzak 1986). Mean densities and masses of *E. lucens* were calculated for each location and an analysis performed to compare the preserved masses (determined with the gut contents) of the mature individuals from each site. The proportion of this species within the earthworm community was also assessed.

**Table 1:** Characteristics of phytosociological groups sampled for earthworms within beech (*Fagetum carpaticum*) forest of the Bieszczady Mountains (Adapted from Kostecka 1988)

Site	Location	Altitude a.s.l (m)	Major tree	Ground cover
I *F. c. festucetosum drymejae*	N 49° 05′ 53″ E 22° 40′ 17″	740	Dominating species is beech, in an admixture of fir and spruce	*Festuca drymeja, Carex pilosa*, in small numbers *Symphytum cordatum*, *Dentaria grandulosa*
II *F. c. typicum*	N 49° 05′ 50″ E 22° 40′ 15″	750	Beech and fir in a sycamore admixture	*Symphytum cordatum*, *Dentaria grandulosa*, *Polystichum braunii*, *D. bulbifera*, *Asperula odorata*
III *F. c. lunarietosum*	N 49° 08′ 54″ E 22° 41′ 11″	650	Beech and sycamore in an admixture of fir and ash	*Lunaria rediviva*, *Urtica dioica*, *Petasites albus*, *Symphytum cordatum*, *Dentaria grandulosa*
IV *F. c. allietosum*	N 49° 03′ 25″ E 22° 45′ 44″	960	Beech and sycamore	*Allium ursinum*

Further visits to these sites in June 2013 and July 2017 permitted the collection of live specimens of *E. lucens* for use in laboratory studies on their life history. These were collected within and below decaying wood at a sub-section of the aforementioned beech-dominated woodland. It was difficult to provide density-related figures (number and mass m^−2^) from these investigations, as mature and immature earthworms, plus cocoons, were all present in the decaying wood which was deliberately targeted for living animals but was not evenly spaced within the wider landscape. All three life stages were collected and taken to laboratories in both Rzeszow and Preston. Initially, these animals were maintained within the material from where they were found, i.e. semi-decomposed beech wood.

A further visit to a site near Lutowiska (49° 17′ 8″ N, 22° 40′ 51″ E) close to the BNP, permitted collection of specimens of *E. lucens* in January 2019. Here the site was relatively easily accessible as it was close to a main road. However, in the forest, snow to a depth greater than 0.5 m had to be cleared from the forest slopes to locate hidden dead beech wood. Once located, material was removed for examination in the laboratory, and a soil pit was dug (0.5 m × 0.5 m) below the dead wood in layers to a depth of 0.8 m. Each 15-cm layer was hand-sorted for earthworms in the field.

### Laboratory work

In one setup, clitellate earthworms were kept for reproduction experiments (*n* = 5 specimens) in plastic containers (30 × 20 × 20 cm) (*n* = 5 replicates) filled with semi-decomposed beech wood substrate and fed regularly from a stock of this material, which was moistened as required. These containers were kept in an air-conditioned room at a constant temperature of 20 ± 0.5 °C. Measurement of earthworm population structure was carried out every 6 months, when the number and mass of individuals (mature and immature) and cocoons were determined.

Further experiments were undertaken to gather data from other life stages. Individual cocoons (some collected from the field and some produced in the laboratory) had masses determined and were incubated individually in Petri dishes on moistened filter papers at 15 °C, in 450-L temperature-controlled incubators (LMS Ltd. Kent) (Lowe and Butt 2005). Cocoons were examined periodically and filter papers re-wetted as necessary. The number of hatchlings per cocoon was recorded, and the mass of each hatchling determined individually. The time taken to hatch could not be assessed accurately as it was compromised by field collection and (in) frequency of collection from breeding systems.

To assess effects of the substrate provided for *E. lucens*, hatchlings were grown at 15 °C. This is a slightly elevated temperature from the field (mean May–Nov soil temp 9.2 °C—Kostecka, 1988), but one which has shown to be acceptable in the laboratory to temperate earthworms. Prior to this, the hatchlings were randomly selected from a collection made over a period of 3 weeks of hatching and maintained refrigerated (to prevent development) at 5 °C in pots of water (Lowe and Butt 2005). For the growth experiments, 3 hatchlings were housed in 350-ml plastic pots (SystemPac, Trowbridge), with pierced, removeable lids (5 replicates per treatment). They were given either field-collected and semi-decomposed wood or under identical conditions, a 1:1 volume ratio of this wood, with dried and then re-wetted horse manure, a reliable food material for earthworms (Lowe and Butt 2005). Sampling occurred every 3 weeks, when the animals had masses determined and were provided with additional substrate, as appropriate. The experiment was terminated after 18 weeks. Comparisons were made of growth rates with the two types of food using *t* tests.

To test the effects of elevated temperature on growth, a further experiment used hatchlings derived as above. With 3 hatchlings per 350-ml pot, replicated ten times, *E. lucens* hatchlings were fed solely with re-wetted horse manure, initially at a lower temperature of 10 °C. After a period of 19 weeks, half of the pots (*n* = 5) were transferred to a higher temperature (15 °C) with the rest remaining at 10 °C. These were all then monitored for a further 15 weeks. Comparisons using *t* tests were made of growth for animals kept under these two temperature regimes.

Each statistical test used was a *t* test, for comparisons of preserved adult masses (1980s data) and for comparisons of hatchling growth. The latter compared growth at either different temperatures (10 vs 15 °C) or with different types of food (wood vs wood and manure). In each case, all appropriate assumptions were met. The statistical package used was Minitab 18 with a critical value of *p* = 0.05.

## Results

### Fieldwork

*E. lucens* was one of 13 earthworm species found within the BNP across the four field sites sampled using Zajonc’s methodology (see Kostecka and Skoczeń 1993 for a full list of earthworm species) and was only present at 2 of the sites investigated where it comprised 1.1 and 4.4% of total numbers of earthworms found in the soil. Adult to sub-adult ratios were in the range 1:2–1:3. Preserved masses (in 4% of formalin, with the gut content) of mature individuals were significantly different (*p* < 0.01) between two sites (site II and site IV) where they were found in the mineral soil during the autumn months (1.00 ± 0.06 vs. 0.69 ± 0.25 g). The mean density of *E. lucens*, averaged across sites was 4.14 ± 3.53 m^−2^ with a mean biomass of 2.21 ± 1.93 g m^−2^. At one of the sites (IV), numbers and associated biomasses of *E. lucens* in the soil increased dramatically from zero present (during May–July), then a monthly stepwise increase to a maximum of 10 m^−2^ in October. At the same site, the density in November was found to be only 1 m^−2^.

Cocoons were found deposited in the dead wood above the soil surface (June and July collections). From field-collected cocoons, mean (± SE) masses were recorded as 50.6 ± 2.9 mg (*n* = 39). Freshly produced cocoons were ovoid, pale yellow and approximately 7 × 4 mm in size. Hatching success rate of field-collected cocoons was 85% when incubated at 15 °C. The mean number of hatchlings per cocoon was 2.9 ± 0.2 (median 3; range 1 to 5; *n* = 27). Mean masses of hatchlings were 35, 16, 10, 7 and 5 mg, for 1, 2, 3, 4 and 5 hatchlings per cocoon, (for *n* = 4, 6, 8, 7, 2 cocoons respectively).

Sampling soil below deadwood under snow, during January, revealed activity of earthworms, but these were individuals of *D. alpina* (20 m^−2^ in the upper 30 cm). Soil temperature was above zero (increasing from 1.4 °C at the surface to 3.8 °C at a depth of 80 cm). Two juvenile *E. lucens* were found within the dead wood collected, as were 12 cocoons. Each cocoon was red in colour and contained a well-developed earthworm. Eleven hatched (92%) within 3–14 days (median 7) of deposition in a Petri dish (as above) at 15 °C. These cocoons had a mean mass of 17 mg and each produced a single hatchling.

### Laboratory work

**Fig. 2:**
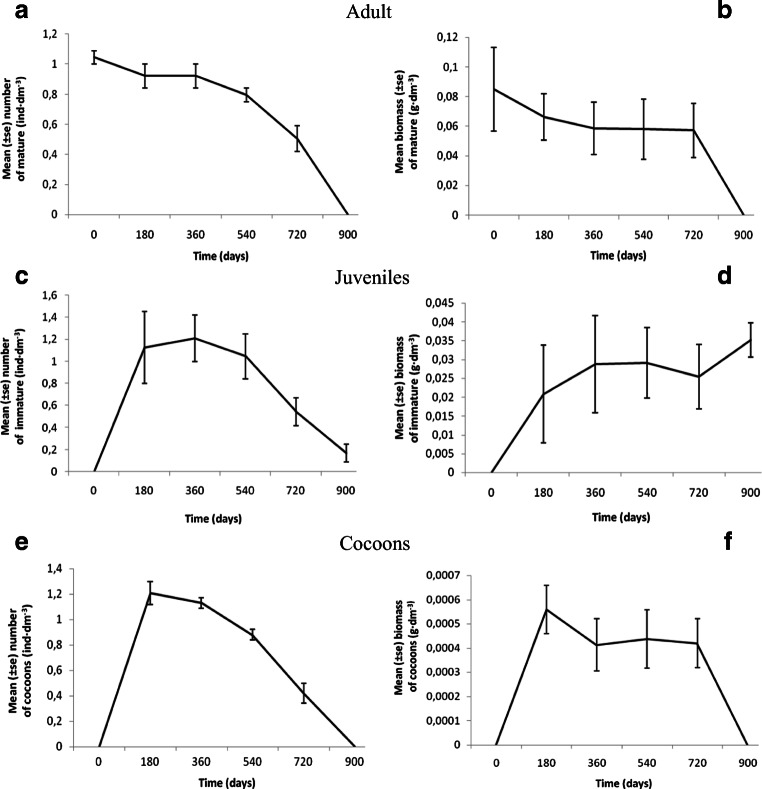
**a**, **c**, **e** The mean number and **b**, **d**, **f** biomass of mature, immature and cocoons respectively, of *E. lucens* in culture over 900 days

Container-scale laboratory culture, with a food supply of rotted wood, allowed maintenance of a dynamic population of *E. lucens* for 720 days (Fig. 2). Thereafter, mature individuals and cocoons were absent and a major decrease in the number of immature individuals was also recorded. The number of mature individuals remained constant from 180 to 360 days (0.92 ± 0.08 ind·dm^−3^) (Fig. 2a) with reproductive level on these sampling days at 0.76 and 0.81 cocoons per mature individual respectively. Despite a significant decrease in the number of mature individuals between 540 and 720 days (by 37%) (Fig. 2a), an average mass was maintained at a similar level from 360 to 720 days at c. 0.06 ± 0.02 g·dm^−3^ (Fig. 2b). Similar trends were observed for immature earthworms and cocoons from 360 to 720 days (Fig. 2c–f). As the number of mature earthworms decreased so did cocoon production (Fig. 2e) and hatchling number (Fig. 2c).

**Fig. 3:**
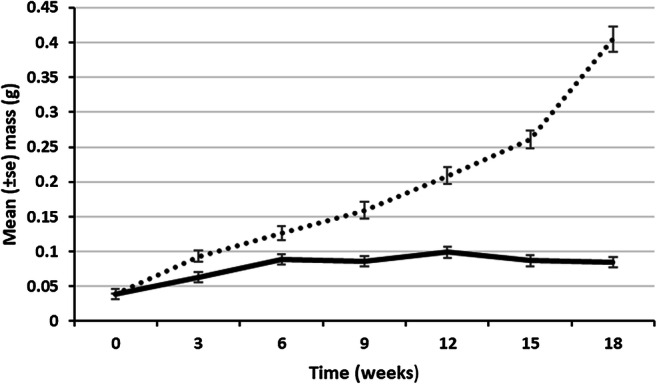
Growth of hatchling *E. lucens* at 15 °C fed with either dead wood only (solid line) or dead wood and horse manure (dotted line)

**Fig. 4:**
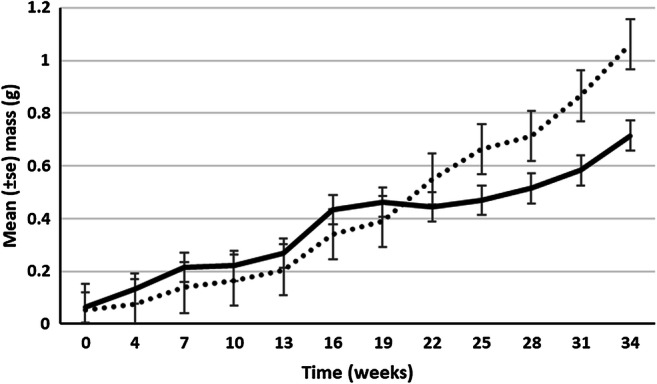
Growth of hatchling *E. lucens* fed with dead wood and horse manure at 10 °C throughout the 34 weeks (solid line) and with a move to 15 °C after 19 weeks (dotted line)

At 15 °C, growth in a substrate composed of a 50:50 ratio of semi-decomposed wood and horse manure led to a significantly greater (*p* < 0.001) increase in biomass than with wood alone (Fig. 3). After 18 weeks, the mean (± SE) masses obtained in the mixture and wood alone were 405 ± 18 and 85 ± 12 mg, respectively. An increase in temperature from 10 to 15 °C also brought about more significant (*p* < 0.05) growth and development (Fig. 4). When moved to an elevated temperature after 19 weeks (at masses of 0.39–0.46 g), *E. lucens* attained a mean mass after 34 weeks of 1.06 ± 0.07 g compared with 0.72 ± 0.12 g when left at 10 °C. The animals that were moved to the higher temperature also showed signs of maturation with tubercular pubertatis present at 34 weeks.

## Discussion

### Fieldwork

It might seem that, in beech-dominated forests of the BNP, *E. lucens* represents a small proportion of the earthworms found, but its apparent distribution and abundance, as revealed from conventional sampling (Zajonc 1970; Butt and Grigoropoulou 2010), is of some interest. A total absence from soil samples collected at 2 sites (I and III) and from the others during the spring and early summer may indicate no more than this earthworm was not in the mineral soil. Previous records (Pes et al. 2016; Szederjesi et al. 2018) and our own collection for laboratory experiments in 2013 and 2017, showed that a large component of the life cycle of this earthworm is enacted within rotting wood above the soil surface. To assess this fully, more than just hand-sorting of soil from a given area plus addition of a vermifuge is required. A more complete survey of earthworms in the beech-dominated forests would sensibly utilize the method proposed by Ashwood et al. (2019) which fully accounts for dead wood in forests when estimating earthworm population density and biomass. Researchers have now come to understand that many epigeic and even endogeic earthworms will exploit resources below the bark of dead wood when conditions allow (Zou et al. 2016, 2018).

An increase in population density, from August to October at one site (IV), was likely to have been the movement of this species back to the mineral soil in preparation for the onset of adverse conditions (winter) when temperatures above the soil drop below freezing point (e.g. recorded as − 2.5 °C in snow and − 8.9 °C in air during January 2019), whereas those within the soil have been shown to be higher. Evidence from a reduced density in the soil during November supports this downward migration as worms may have burrowed significantly deeper and been unresponsive to extraction techniques.

Further sustained work would usefully examine what happens to *E. lucens* and co-occurring earthworms during the winter months in forest soils, when detailed field sampling has not been undertaken due to deep snow and restricted access. Are these earthworms deep in the soil, as seen for adults of other species, such as *Lumbricus terrestris* and *Aporrectodea caliginosa* in agro-ecosystems when temperatures fall (Nuutinen and Butt 2009), or possibly coiled in frozen layers? In addition, which life stages can persist within the upper soil layers or in rotten wood over winter? Our preliminary observations suggest that juveniles and cocoons can overwinter in dead wood at the soil surface. Smaller cocoons were located in January compared with summer collection (mean masses 17 mg and 51 mg respectively). The winter-collected cocoons also produced just a single hatchling. This could be important and part of a specific survival strategy or simply relate to sampling from different locations. Monthly collection at a number of sites throughout the year would seek to address this.

### Laboratory work

This species can be maintained in the laboratory, but there is a need to ensure that field-collected individuals are initially provided with a substrate collected from the forest. Container-scale experimentation showed that the complete life cycle was achieved, and a steady (density-dependent) state was reached. However, there was a need to re-provision the system with new substrate at periodic (6 months) intervals. After 360 days, a deterioration in population size was recorded, despite substrate re-provision. This could have been a function of the relatively high temperature (20 °C) at which the animals were maintained, leading to reproductive fatigue (Butt 1997) or perhaps showed that the substrate, collected at the start of the experiment had been further biologically degraded during storage and no longer had the nutritional value previously provided. Under culture conditions, it is necessary to try and find alternative (standard) food sources, if possible, and adapt cultures to these (Lowe and Butt 2005). The smaller, more controllable, pot-related work, at lower temperatures with introduction of horse manure as a feedstock, seems to support these suggestions. Although dead wood may be the natural food of this species, horse manure was shown to be a superior source of nutrition, as growth was enhanced by its use.

The use of a 1:1 volume ratio of wood and horse manure led to significantly increased juvenile growth rates at 15 °C suggesting that, e.g. the higher C:N ratio of the horse manure was important and not deleterious to the growth of this species. Similar observations have been made for other species (e.g. Lowe and Butt 2005). The important point here is to introduce the earthworms to an enriched diet slowly or by proportion and more easily at a lower temperature. Increased growth rates when temperatures were elevated (here from 10 to 15 °C) was not unexpected for earthworms in general (Lowe and Butt 2005) but can only work within the physiological tolerances for the given species. Here these were not explored to the limit, but it is something that could be investigated further.

## Conclusions

The aims of this work have been partially met. Field data of preserved adult *E. lucens* have been presented and compared with live animals collected more recently. New information on cocoons and emerging hatchlings has been derived from collections in rotting wood during the summer. Further (small scale) collection in winter has also revealed part of the behavior within the life cycle of this species in mountain forests. Nevertheless, there is still much to be learned, and some of the questions posed above could usefully be addressed by undertaking seasonal sampling, throughout the year by searching quantitatively in both mineral soil and in deadwood.

Life cycle data from the laboratory showed that this species can be maintained and will thrive on an artificial diet of manure, permitting aspects of life stages to be investigated. Much more could be undertaken here to determine if this species might have attributes, as yet unexplored. These could potentially involve organic matter management, but breeding of this species might have benefits for ecosystem restoration in areas where forests are degraded or found to have changing earthworm communities, as exotic species become established (e.g. Pop and Pop 2006).
